# DEPDC5: Modeling of the Two-Hit Wonder in Cortical Organoids

**DOI:** 10.1177/15357597261464330

**Published:** 2026-07-13

**Authors:** Erin L. Heinzen

**Affiliations:** Division of Pharmacotherapy and Experimental Therapeutics, Eshelman School of Pharmacy, University of North Carolina at Chapel Hill, Chapel Hill, NC, USA; Department of Genetics, School of Medicine, University of North Carolina at Chapel Hill, Chapel Hill, NC, USA

## Abstract

**Mosaic Human Cortical Organoids Model 1 mTOR-Related Focal Cortical Dysplasia 2 Through DEPDC5 Deletion**

Maletic M, Bizzotto S, Ribierre T, Guerdoud K, Raoux C, Doladilhe M, Dalle C, Picard F, Baulac S. Brain 2026:awag086. doi: 10.1093/brain/awag086. Online ahead of print. PMID: 41789478

Focal cortical dysplasia type II (FCDII), a major cause of pediatric drug-resistant focal epilepsy, results from brain somatic variants in the mechanistic target of rapamycin (mTOR) pathway genes, including germline and somatic second-hit loss-of-function variants in the mTOR repressor DEPDC5. Here, we present a proof-of-concept model of DEPDC5 two-hit inactivation mosaicism using patient-derived human cortical organoids (hCOs). Mosaic hCOs displayed increased mTOR activity that was rescued by the mTOR inhibitor rapamycin. Mosaic hCOs also exhibited dysmorphic-like neurons and enhanced neuronal excitability, recapitulating key FCDII pathology hallmarks. Single-cell transcriptomics across 3 developmental stages revealed aberrant differentiation trajectories leading to premature upper-layer neuron generation, upregulated Notch and Wnt signaling pathways in neural progenitors, and altered expression of synaptic- and epilepsy-associated genes in excitatory neurons. In addition, we identified cell-autonomous alterations in metabolism and translation in mosaic DEPDC5 two-hit hCOs. This study provides novel insights into how DEPDC5 deficiency perturbs human corticogenesis, highlighting that mosaic bi-allelic inactivation of the gene is necessary for FCDII pathogenesis.

## Commentary

In 2013, two studies using linkage analysis and exome sequencing identified loss-of-function variants in *DEPDC5* as a cause of familial focal epilepsy.^1,2^ *DEPDC5* encodes a core component of the GATOR1 complex, a negative regulator of the mechanistic target of rapamycin complex 1 (mTORC1) pathway that inhibits cell growth in the absence of nutrients.

Individuals with *DEPDC5*-associated focal epilepsy exhibited remarkable phenotypic heterogeneity within and across families. It was also unclear how a germline variant present in all cells causes focal seizures. These observations led to the hypothesis that a second genetic variant may arise during embryonic brain development that could explain the phenotypic variability and seizure localization observed in individuals with *DEPDC5* epilepsy. This “two-hit” mechanism, first conceptualized in the cancer field, was later implicated in epilepsy following identification of second-hit variants in *TSC1* and *TSC2* with tuberous sclerosis complex, and the first hit is spontaneous or inherited loss-of-function of one allele.^3^ Consistent with this hypothesis, second hit mutations were identified in brain epileptogenic tissue therapeutically resected from two individuals with a pathogenic germline *DEPDC5* variant. These second hits were found on opposing alleles consistent with bi-allelic inactivation.^4,5^

Neuropathological features associated with focal cortical dysplasia type II (FCDII), including dysmorphic neurons, are often found in resected brain tissue from individuals with *DEPDC5* epilepsy. Neurons with bi-allelic inactivation of DEPDC5 have been shown to be enriched in dysmorphic neuron populations.^6^ Rodent models have further demonstrated that spontaneous seizures are associated with bi-allelic loss of DEPDC5, whereas heterozygous loss leads to no or only modest increases in seizure susceptibility.^7,8^

Despite these advances toward understanding *DEPDC5* pathophysiology, there remain significant gaps in how cells with mono- and bi-allelic hits in DEPDC5 alleles interact in the developing human brain. To begin to address this, Maletic et al^9^ established an elegant *in vitro* system that uses patient-derived human cortical organoids (hCOs) to model mono- and bi-allelic mosaicism in the developing human brain. They used human-induced pluripotent stem cells (hiPSCs) derived from an affected child harboring a heterozygous germline loss-of-function *DEPDC5* variant, and an unaffected sibling without the loss of function variant. Using CRISPR-Cas9, they then introduced the same pathogenic allele into the unmutated (wildtype) *DEPDC5* allele, along with a fluorescent reporter, in the affected child's cells. hiPSCs from the unaffected brother were used as the control. Genetically homogenous hCO were generated from the control cell line and heterozygous (DEPDC5^+/−^) hiPSC lines. Mosaic organoids modeling two-hit mosaicism were generated by mixing of mono- (DEPDC5^+/−^) and bi-allelic (DEPDC5^−/−^) hiPSC lines to create mosaicism.

The authors demonstrate that their hCO model system recapitulated the known mechanism of action by showing that only bi-allelic loss resulted in constitutive mTOR activation under nutrient-deprived conditions. DEPDC5^–/–^ neurons in their model exhibited enlarged somas and neurofilament accumulation, also consistent with dysmorphic neurons found in the human condition. These findings provide additional validation of previously published work in human tissue and rodent models that bi-allelic, but not mono-allelic, inactivation drives both mTOR hyperactivation and morphological abnormalities. Furthermore, these results establish the novel organoid model system as a valuable research tool for in-depth studies of the molecular mechanisms of *DEPDC5* epilepsy.

The authors employed longitudinal single-cell RNA sequencing (scRNA-seq) across three developmental stages to analyze cellular composition, gene expression, signaling pathways, morphology, and network activity. Both pure heterozygous and mosaic organoids showed premature differentiation as evidenced by an increased proportion of upper-layer excitatory neurons. Precocious differentiation was also supported by reduced neural rosette density in both the heterozygous and mosaic developing hCOs compared to control. Computational analyses evaluating the ratio of unspliced pre-mRNA to mature spliced mRNA of cell type marker levels as a way to predict the future state of individual cells suggests that DEPDC5 loss accelerates the transition from progenitors to upper-layer neurons. Differential expression analyses suggest that this may be driven by dysregulation of Notch and Wnt signaling pathways, although additional studies will be needed to establish if the transcriptional changes are the cause or effect of cellular composition changes observed.

The *DEPDC5* mosaic hCO model also captured transcriptional changes in excitatory neurons that may drive the hyperexcitability phenotype. Specifically, differential expression analyses revealed that excitatory neurons from both heterozygous and mosaic mature organoids exhibited increased expression of epilepsy-related genes, including *KCNQ3*, *SCN2A*, and *CACNA1A*. Synaptic genes were also enriched among differentially expressed genes in mosaic organoids.

A strength of the established mosaic model is its capacity to distinguish between cell-autonomous from non-cell-autonomous effects. Applying scRNA-seq further allowed the authors to resolve cell-type-specific effects, showing that *DEPDC5*^−/−^ excitatory neurons exhibited transcriptional profiles consistent with cell-autonomous metabolic and translational dysregulation that may contribute to dysmorphic neuron formation and hyperexcitability.

Human cortical organoid models have emerged as a powerful platform for studying the cellular and molecular underpinnings of neurodevelopmental disorders. These models provide a valuable platform for studying very early human cortical differentiation, a developmental window that is difficult to assess in humans or rodent models. By establishing an organoid platform for the study of mosaic *DEPDC5* epilepsy, the authors have created a foundation upon which deeper mechanistic questions can begin to be investigated. The authors provide early mechanistic insight into several key areas ripe for deeper characterization, including linking loss of DEPDC5 to specific cellular and molecular alterations that drive early brain development phenotypes.

Among the most significant weaknesses is that all work was performed in a single patient-derived cell line, raising concerns about reproducibility across independent patient lines and the generalizability of the results. Compounding this, the study employed limited sampling across developmental time points, restricting the resolution with which the temporal dynamics can be characterized and reducing power to detect disease-relevant changes. A further concern relates to the introduction of the second hit in hiPSCs which does not recapitulate the timing of second hits thought to occur during embryonic brain development. The study also attempted to link cell-type-specific transcriptional changes to the genetic variants present in the organoids. Unfortunately, the high variability of these signals makes it difficult to draw firm conclusions about the biological significance of these findings.

It is also important to acknowledge broader constraints inherent to cortical organoid systems. The hCOs differentiation procedure used in this study lacks the full complement of cell types found in the mature human brain, notably oligodendrocytes, and does not recapitulate the circuitry that is critical to human brain development. While advances in differentiation protocols that allow for earlier development of oligodendrocytes and assembloid systems that fuse multiple brain regions offer partial solutions, organoid models will never fully replicate the complexity of an intact human or rodent brain.

While the study carries methodological limitations commonly associated with early-stage organoid studies, this does not diminish its core achievement. The hCO platform faithfully recapitulates key phenotypes associated with *DEPDC5* epilepsy that are observed in patients. This model provides novel early mechanistic insight in *DEPDC5* pathophysiology that can be expanded on in future work to drive forward the vision of precision therapy for genetic epilepsies. Moreover, the experimental framework developed here can serve as a template for modeling other mosaic brain diseases using hCOs.
